# Altered composition and phenotype of mucosal-associated invariant T cells in early untreated rheumatoid arthritis

**DOI:** 10.1186/s13075-018-1799-1

**Published:** 2019-01-05

**Authors:** Hester Koppejan, Diahann T. S. L. Jansen, Marjolijn Hameetman, Ranjeny Thomas, Rene E. M. Toes, Floris A. van Gaalen

**Affiliations:** 1 Department of Rheumatology, Leiden University Medical Center, PO Box 9600 (Zone C1-R), Albinusdreef 2, 2233 ZA Leiden, the Netherlands; 2 Diamantina Institute, Translational Research Institute, University of Queensland, Woolloongabba, QLD Australia; 3 Department of Gastroenteroloy and Hepatology, Erasmus MC University Medical Center Rotterdam, Rotterdam, the Netherlands

**Keywords:** Rheumatoid arthritis, Spondyloarthritis, Mucosal-associated invariant T cells, CD161

## Abstract

**Background:**

Mucosal-associated invariant T (MAIT) cells are innate-like T cells that recognise bacterial metabolites presented by MHC class I-related protein 1 (MR1). Bacterial dysbiosis has been implicated in auto-inflammatory disease development. We investigated MAIT cells in early, untreated rheumatoid arthritis (RA) and spondyloarthritis (SpA) patients.

**Methods:**

Blood and synovial fluid mononuclear cells obtained from patients (SpA/RA) and controls were stimulated with fixed *Escherichia coli* to provide MAIT ligand. Cells were analysed by flow cytometry and MAIT cells were identified by MR1-5-OP-RU tetramers. Synovial biopsies were studied by confocal microscopy.

**Results:**

Peripheral and synovial CD3^+^ MR1-tet^+^ MAIT cell frequencies were comparable in all groups. MAIT cells were detected in RA and SpA synovium based on CD3, CD161 and Vα7.2 expression. Peripheral RA MAIT cells were mostly CD4^+^ (controls 8.3%, SpA 12.3%, RA 52.6%; *p* < 0.001) and CD161 expression was strongly reduced (control mean fluorescence intensity (MFI) = 2348, SpA MFI = 2219, RA MFI = 226; *p* < 0.001). MAIT cells were hyporesponsive, shown by minimal upregulation of CD25 and CD69 to *E. coli* stimulation (control, CD25 MFI = 177, CD69 MFI = 1307; SpA, CD25 MFI = 95, CD69 MFI = 1257; RA, CD25 MFI = 0, CD69 MFI = 467; *p* < 0.001 and *p* = 0.01 respectively).

**Conclusion:**

In early untreated RA patients, the peripheral MAIT cell composition was altered, with reduced levels of CD161 expression, and cells were hyporesponsive to stimulation. MAIT cell dysfunction may provide a link between the microbiome and development of RA.

**Electronic supplementary material:**

The online version of this article (10.1186/s13075-018-1799-1) contains supplementary material, which is available to authorized users.

## Background

Mucosal associated invariant T (MAIT) cells are innate-like T cells that express a semi-invariant T-cell receptor (Vα7.2-Jα33 in humans, Vα19-Jα33 in mice). They represent up to 10% of total peripheral T cells. The T-cell receptor (TCR) of MAIT cells recognises riboflavin metabolites (vitamin B2 derived) in the context of the non-polymorphic, highly conserved MHC class Ib-related protein (MR1) [1]. These metabolites are synthesised by numerous yeast and bacteria, and therefore MAIT cells are thought to play a role in bacterial immunity. MAIT cells are equipped with a wide range of interleukin and chemokine receptors and mostly reside in the liver and the mucosal tissue [2]. Upon activation, MAIT cells exert their effector function mainly through the production of TNF, although production of IFN-γ, Granzyme B and IL-17 has also been described [3, 4].

For over 30 years, different studies described a possible link between gut immunity and axial spondyloarthritis (SpA). Additionally, the notion is emerging that microbial dysbiosis and pathogens are also instrumental in the development of rheumatoid arthritis (RA) [5]. Together, development of both RA and SpA has been linked to microbial triggers, although a single disease-causing microbe has not been identified.

MAIT cells have been described in SpA and RA [6–8]. While the frequency of Vα7.2^+^CD161^hi^ MAIT cells were observed to be reduced in peripheral blood of RA patients, as compared to SpA patients, it was not clear whether this difference was disease or treatment related as samples from treated patients with longstanding disease were studied.

To assess a potential role of MAIT cells in early disease, we analysed the frequency and activity of MAIT cells in untreated early RA patients, untreated SpA patients and controls.

## Patients and methods

### Patient material

RA blood samples (*n* = 10) were collected as part of the Leiden Early Arthritis Clinic (EAC) cohort study of patients with arthritis of recent onset [9]. Axial SpA and control blood samples (*n* = 12 and *n* = 10 respectively) were collected as part of the SPondyloArthritis Caught Early (SPACE) cohort study [10]. Synovial fluid (SF) samples were collected in the outpatient clinic from patients with active RA (*n* = 8) and SpA (*n* = 8) at the Department of Rheumatology, Leiden University Medical Centre, Leiden, the Netherlands. Peripheral and synovial mononuclear cells (PBMCs/SFMCs) were isolated by Ficoll-Paque gradient centrifugation and cryopreserved until use. Patient characteristics are summarised in Additional file 1: Table S1. Notably, for PBMC samples all participants had a short symptom duration and had not used oral corticosteroids, biologics or DMARDs to treat arthritis (one SpA patient had taken low-dose methotrexate for psoriasis). Patients donating SFMC samples had comparable symptom duration but, in contrast to PBMC donors, all RA patients and half of the SpA patients used DMARDs. Synovial biopsies were obtained from SpA and RA patients undergoing arthroscopy of the knee because of arthritis. Tissue biopsies were snap-frozen and stored until use. Studies were approved by the LUMC ethical committee and all patients provided written informed consent.

### In-vitro stimulation

PBMCs and SFMCs were thawed and plated at 2.5 × 10^5^–5 × 10^5^ cells/well (U-bottom 96-well plate, Costar; Sigma) in Iscove’s Modified Dulbecco’s Medium (IMDM) (Lonza Bioresearch) supplemented with 8% heat-inactivated fetal calf serum (FCS), penicillin/streptomycin (100 U/ml) and 2 mM Glutamax. *Escherichia coli* strain DH5α was fixed in 1% paraformaldehyde (LUMC Pharmacy), washed and stored at 4 °C until use. Cells were stimulated with fixed *E. coli* at a multiplicity of infection (MOI) of 6 and incubated overnight without additives.

### Flow cytometry

Cells were stained for surface markers: CD3-FITC (SK7), CD8α-AF700 (RPA-T8), CD14-PB (M5E2) and CD69-PECF594 (FN50) (BD Biosciences, San Jose, CA, USA); CD3-BV605 (SK7), CD4-APC-Cy7 (SK3), CD19-BV421 (HIB19) and CD25-PerCPCy5.5 (M-A251) (Biolegend, San Diego, CA, USA); CD161-APC or CD161-FITC (191B8; Miltenyi Biotech, Bergisch Gladbach, Germany); and an MR1 tetramer (provided by Prof. Jamie Rossjohn) which consisted of 5-OP-RU-loaded MR1 monomers [11] conjugated to Streptavidin-PE (eBioscience, San Diego, CA, USA). In contrast to regular buffers, tetramer FACS staining was performed using PBS supplemented with 2% FCS (no azide). Isotype controls were used for the expression of CD25 and CD69. DAPI (200 mM; Molecular Probes) and LIVE/DEAD® Fixable Aqua Dead Cell Stain Kit (Molecular Probes by Life Technologies; ThermoFisher Scientific, Waltham, MA, USA) were used to define live cells in PBMC and SFMC samples respectively. All samples were acquired on an LSRFortessa (BD) and analysed using FlowJo v10 (TreeStar, Ashland, OR, USA).

### Confocal microscopy

Snap-frozen synovium biopsies were cut into 5-μm slices and mounted onto Menzel-Gläser SuperFrost slides and stored at − 20 °C. Briefly, slides were thawed, fixed in cold acetone, blocked with TNB (0.1 M Tris–HCl, 0.15 M NaCl, 0.5% blocking agent; Roche, Basel, Switzerland), stained with primary and secondary antibodies separately and embedded with ProLong Gold Antifade (Life Technologies). The panel included rat anti-human CD3 (CD3-12; Biorad, Hercules, CA, USA), mouse anti-human Vα7.2 (IgG1, 3C10; Biolegend), rabbit anti-human CD20 (RB-9013-P; Life Technologies, ThermoFisher Scientific), mouse anti-human CD161 (IgG2a, 191B8; Miltenyi) and Hoechst 33342 (50 μg/ml; Life Technologies). Secondary antibodies included goat anti-rat-AF594, goat anti-mIgG1-AF488, goat anti-rabbit-AF647 and goat anti-mIgG2a-AF546 (Life Technologies). Acquisition of images was done on a Leica TCS SP8 X White Light Laser with a 63× oil objective using Leica Acquisition Suite X software (Leica Microsystems B.V, Amsterdam, the Netherlands).

### Statistics

Data analyses were performed in GraphPad Prism v7 (GraphPad Software, La Jolla, CA, USA), using either the Kruskal–Wallis test (> 2 groups) or Mann–Whitney *U* tests (two groups). Statistical significance was considered when *p* < 0.05.

## Results

**Fig. 1:**
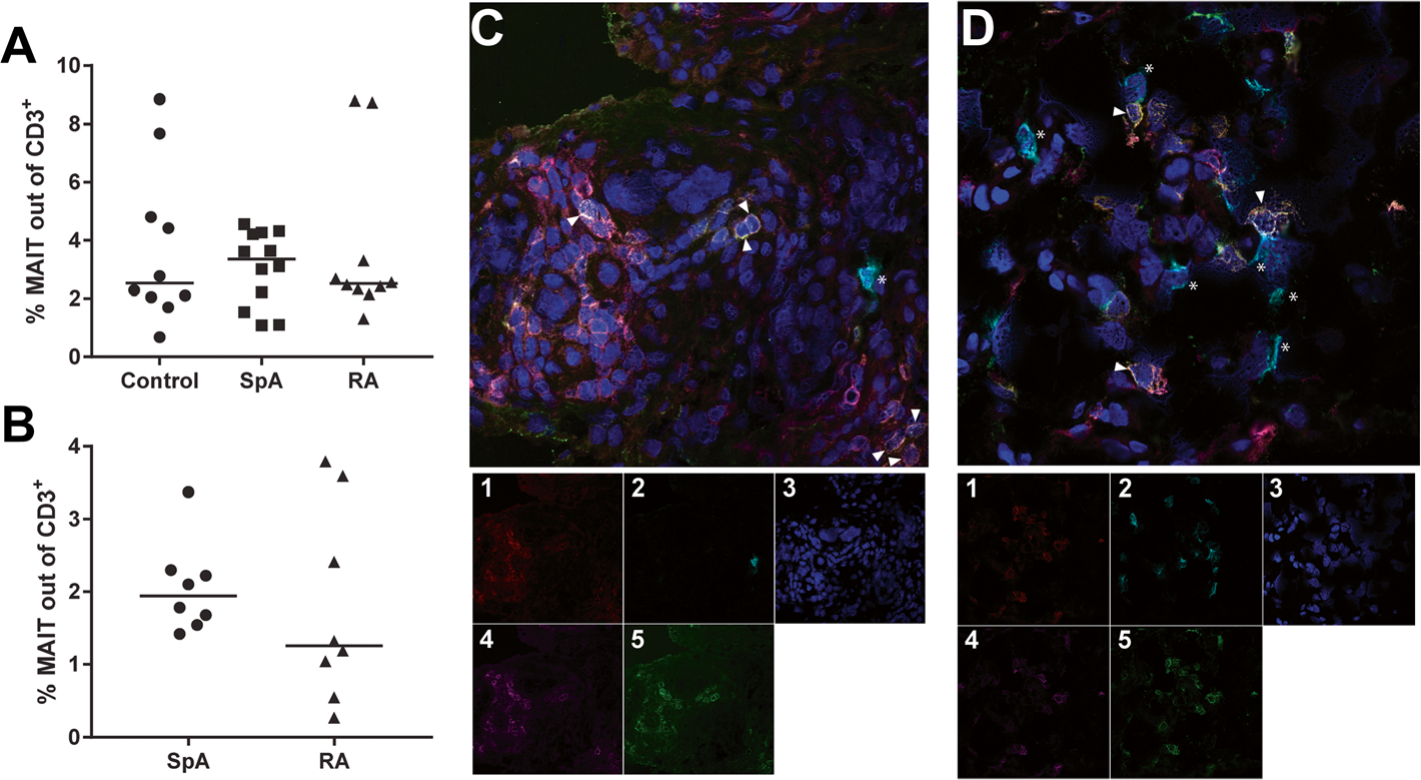
MAIT cells present in peripheral blood, synovial fluid and synovium. **a, b** MAIT cells present in both blood (PB, a) and synovial fluid (SF, b) and frequencies of total MAIT cells (CD3^+^MR1-tet^+^) in PB and SF are comparable between groups. **c, d** Representative images of SpA (c) and RA (d) synovium confirming MAIT cells are also present within synovium of SpA and RA patients. MAIT cells, triangles; B cells, asterisks. MAIT cells defined as CD3^+^CD161^+^Vα7.2^+^ and B cells as CD19^+^. (1) CD3; (2) CD19; (3) Hoechst; (4) CD161; (5) Vα7.2. MAIT mucosal-associated invariant T, RA rheumatoid arthritis, SpA spondyloarthritis

We determined the frequency of MAIT cells in SpA and RA patients and controls in both peripheral blood and synovial fluid using 5-OP-RU-loaded MR1 tetramers [11]. MAIT cells were analysed by flow cytometry and defined as CD3^+^MR1 tetramer^+^ (MR1-tet^+^) (gating strategy shown in Additional file 2: Figure S1). The frequency of CD3^+^MR1-tet^+^ cells in peripheral blood of controls and early, untreated SpA and RA patients was comparable in all groups (Fig. 1a; median controls 2.5%, SpA 3.4%, RA 2.5%; *p* = 1.0). MAIT cells were also present in SF. Frequencies were similar in both groups, although the values showed more variation in RA compared to SpA (Fig. 1b; median SpA 1.9%, RA 1.3%; *p* = 0.4). Using CD3 combined with Vα7.2 and CD161, the presence of MAIT cells was analysed by confocal microscopy in synovial tissue. As shown in Fig. 1c, d, MAIT cells are also present in synovial tissue of SpA and RA patients. No quantitative SpA vs RA comparisons were performed due to the small sample size.

**Fig. 2:**
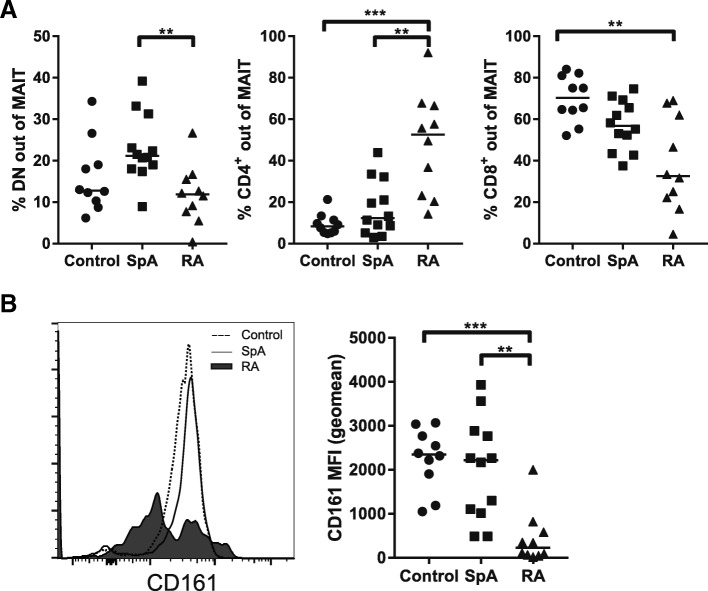
Shift in CD4^+^/CD8^+^ MAIT cell subsets and low CD161 expression in early, untreated RA patients. **a** MAIT cells divided based on CD4 and CD8 expression. Whereas MAIT cells are generally mostly CD8^+^, in early RA patients most MAIT cells are CD4^+^, indicating a shift within the MAIT cell population. Median double-negative MAIT cells (DN): controls 12.8%, SpA 21.2%, RA 11.2%. Median CD4^+^ MAIT cells: controls 8.4%, SpA 12.3%, RA 52.6%. Median CD8^+^ MAIT cells: controls 70.3%, SpA 56.8%, RA 32.6%. **b** CD161 expression lower in early RA patients compared to controls and SpA patients, both ex vivo and upon stimulation (based on MFI). Median CD161 MFI unstimulated MAIT cells: controls 2348, SpA 2219, RA 226. Median MFI upon stimulation: controls 2134, SpA 1844, RA 482.5. **p ≤ 0.01; ***p ≤ 0.001. MAIT mucosal-associated invariant T, MFI mean fluorescence intensity, RA rheumatoid arthritis, SpA spondyloarthritis

We determined the composition of the MAIT cell population based on CD4/CD8 expression: double negative (DN), CD4^+^ or CD8^+^ MAIT cells. Only a few MAIT cells were DN in RA patients and controls, but these were increased in early SpA patients (Fig. 2a left panel; median controls 12.8%, SpA 21.2%, RA 11.9%; *p* = 0.0079, SpA vs RA *p* = 0.0069). MAIT cells predominantly express CD8, which is in line with the CD8 expression observed in controls and early SpA patients (Fig. 2a right panel; median controls 70.3%, SpA 56.8%). In contrast, in early, untreated RA patients the distribution of MAIT cells shifted from predominantly CD8^+^ (median RA 32.6%) to mainly CD4^+^ MAIT cells (Fig. 2a middle panel; median CD4^+^ MAIT controls 8.3%, SpA 12.3%, RA 52.6%; *p* < 0.001, controls vs RA *p* < 0.001, SpA vs RA *p* = 0.0073). A similar analysis was performed for SFMC samples of SpA and RA patients with established disease, although we observed no differences (Additional file 3: Figure S2A).

Decrease of CD161 expression has been implicated in poor MAIT cell function [12, 13]. Therefore, as a proxy for function, we also investigated CD161 expression by CD3^+^MR1-tet^+^ cells (geo mean fluorescence intensity (MFI); Fig. 2b). CD161 was expressed by almost all CD3^+^MR1-tet^+^ cells and the level of expression was similar between controls and early SpA patients (median control MFI = 2348, SpA MFI = 2219). However, MAIT cell CD161 expression was strikingly lower in early untreated RA patients (RA MFI = 226; *p* = 0.0003, controls vs RA *p* = 0.0006, SpA vs RA *p* = 0.0031). In the case of SFMCs, we observed no differences in CD161 expression by SpA and RA MAIT cells (Additional file 3: Figure S2B).

**Fig. 3:**
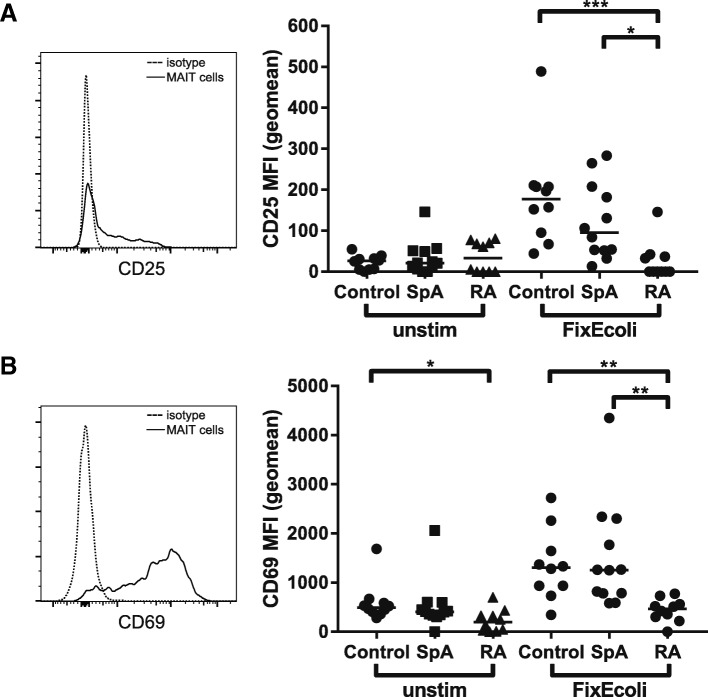
MAIT cells hyporesponsive upon fixed *Escherichia coli* stimulation in early untreated RA patients. PBMCs stimulated overnight by fixed *E. coli* (MOI = 6). CD25 (a) and CD69 (b) upregulation used as a proxy for activation. **a** Basal expression of CD25 relatively low and comparable in all groups. Stimulation by fixed *E. coli* induced upregulation of CD25 in controls (median MFI = 177) and SpA patients (median MFI = 95.15). RA MAIT cells barely responded to fixed *E*. *coli* stimulation (median CD25 MFI = 0). **b** Basal expression of CD69 lower in RA patients (median RA MFI = 196.5 vs control MFI = 495.7 vs SpA MFI = 405.1). Fixed *E*. *coli* stimulation clearly increased CD69 expression in controls (median MFI = 1307) and SpA patients (median MFI = 1257). CD69 upregulation in RA patients was significantly lower compared to controls and SpA (median RA MFI = 466.6). *p ≤ 0.05; **p ≤ 0.01; ***p ≤ 0.001. MAIT mucosal-associated invariant T, MFI mean fluorescence intensity, RA rheumatoid arthritis, SpA spondyloarthritis

We investigated the response of MAIT cells to bacterial ligand by stimulating PBMCs overnight with fixed *E. coli*. Upregulation of CD25 and CD69 MFI was used to determine activation upon stimulation (isotype controlled). Basal CD25 expression was equivalent in all groups and increased upon stimulation (Fig. 3a). Intriguingly, the CD25 did not increase in MAIT cells from early, untreated RA patients, suggesting that either RA MAIT cells or CD4^+^ MAIT cells react poorly to *E*. *coli* ligand (median control MFI = 177, SpA MFI = 95.2, RA MFI = 0; *p* = 0.0004, controls vs RA *p* = 0.0005, SpA vs RA *p* = 0.0110). Basal CD69 expression was lower in RA patients compared to controls (Fig. 3b; median control MFI = 495.7, SpA MFI = 405.1, RA MFI = 196.5; *p* = 0.01, controls vs RA *p* = 0.01). After exposure to fixed *E*. *coli*, CD3^+^MR1-tet^+^ cells from controls and SpA patients strongly increased CD69 expression (median control MFI = 1307, SpA MFI = 1257), while CD3^+^MR1-tet^+^ cells from RA patients did not (median RA MFI = 466.6; *p* = 0.0004, controls vs RA *p* = 0.0021, SpA vs RA *p* = 0.0014).

## Discussion

In early untreated RA patients, the peripheral MAIT cell composition was altered based on CD4^+^ and CD8^+^ MAIT cell frequencies. Furthermore, MAIT cells expressed lower levels of CD161 in early untreated RA patients. Lastly, the CD25 and CD69 response to fixed *E. coli* stimulation was reduced in early untreated RA MAIT cells compared with control or SpA MAIT cells.

Most studies on MAIT cells include Vα7.2 and CD161 to define MAIT cells by flow cytometry [6–8, 14]. Using a the MR1-tetramer loaded with 5-OP-RU [11], we specifically stained MAIT cells irrespective of their Vα usage and discovered that MAIT cell frequencies are similar in all three groups (CD3^+^MR1-tet^+^ cells). Moreover, our results indicate that MAIT cells express low levels of CD161 in early, untreated RA patients. In contrast to our data, some reports suggest that MAIT cell frequencies drop in RA [6–8, 15]. This discrepancy could be explained by the fact that CD161 is often used as part of the MAIT cell gating strategy. This is also supported by Gherardin et al. [16] Their work showed that combining Vα7.2 and CD161 will identify the majority of MAIT cells; however, they emphasise that not all Vα7.2^+^CD161^hi^ cells are MAIT cells compared to MR1–5-OP-RU tetramer staining, especially the CD4^+^ MAIT cell subset. Our data showed that CD161 is reduced in early, untreated RA patients, combined with the observation that CD3^+^MR1-tet^+^ cell frequencies were similar in patients and controls, suggests that the MAIT composition and phenotype is altered rather than an actual loss of MAIT cells.

A change of MAIT cell composition in RA has been reported before based on the Vα7.2^+^CD161^+^ MAIT cell definition [6, 7]. Most of the patients included in these studies had either a long symptom duration (mean duration 6 years) and could potentially be different compared to early RA patients. Additionally, patients received various kinds of disease-modifying anti-inflammatory treatments (including steroids) and therefore a treatment effect cannot be ruled out. Our current study confirms the change in MAIT cell composition reported by others with the use of a MAIT-specific tetramer. Notably, the samples used in the current report were obtained from treatment-naïve patients, which therefore rules out a treatment effect.

The relevance of CD4 and CD8 on MAIT cells is not completely clear. A recent study by Kurioka et al. [17] investigated the phenotype and functions of different MAIT cell subsets. Interestingly, although CD4^+^ MAIT cells were responsive to MR1-dependent stimuli, their T-helper 1 effector functions were reduced. Kurioka et al. were able to relate this to differences in transcription factor expression compared to CD8^+^ and DN MAIT cells. Perhaps the shift from CD8^+^ MAIT to CD4^+^ MAIT we observed in early untreated RA patients could explain the reduced response to *E. coli* stimulation. Finally, Gherardin et al. [16] highlight differences between MAIT cell subsets, including differences in surface markers and cytokine production. Their work showed that CD4^+^ MAIT cells produce 5-fold more IL-2 upon PMA/ionomycin stimulation. Our study only used surface makers as a proxy for activation upon fixed *E. coli* stimulation. This stimulation is less harsh compared to PMA/ionomycin and could therefore result in more subtle differences between groups. For future studies it would be interesting to measure cytokine production of the different MAIT cell subsets to investigate whether these are also altered in early RA in relation to the changes in CD4/CD8 MAIT cell balance.

It is conceivable that MAIT cells become exhausted due to continuous stimulation during chronic inflammation, which may provide an explanation for poor cell activation upon stimulation. In particular, it has been described that loss of CD161^++^ MAIT cells could impact mucosal defence [12]. Leeansyah et al. [13] determined that MAIT cell CD161 levels dropped upon prolonged exposure to *E. coli*. Moreover, reduced CD161 levels upon HIV infection recoverd upon anti-viral treatment. Furthermore, Aldemir et al. [18] showed that, upon interaction with its ligand ‘lectin-like transcript 1’ (LLT1), CD161 was downregulated in NK cells. Germain et al. [19] described that LLT1 is expressed by activated antigen presenting cells and ligation inhibited NK cell effector functions. These observations suggest that CD161 is downregulated in peripheral MAIT cells due to ligand interaction. Additionally, chronic activation could potentially lead to exhaustion, which would fit with our findings showing RA MAIT cells were hyporesponsive upon activation. Cho et al. [6] reported that PD-1 expression was not elevated in RA MAIT cells. As their MAIT cell definition is based on high expression of CD161, their analysis does not include CD161^low^ MAIT cells, which are potentially dysfunctional or hyporesponsive MAIT cells, and those may have elevated levels of PD-1. We have no additional data to confirm exhaustion (such as PD-1 expression); however, low CD161 expression is compatible with chronic activation and could be suggestive for diminished effector function.

We observed no differences in SFMC samples, which were all obtained from patients with established disease. Moreover, SFMC and PBMC samples are non-paired, obtained from different individuals. Consequently, it is difficult to conclude whether the CD161 expression is equally low in both groups or whether levels have recovered upon treatment compared to peripheral MAIT cells. Keller et al. [20] recently showed that MR1 molecules can present metabolites of diclofenac (activating) and methotrexate (non-activating), both used in the treatment of RA. Therefore, it cannot be ruled out that the MAIT cells in SFMCs used in our study were altered by medication. Future analysis of SFMCs and synovium of early, untreated patients will give more insight into this matter.

## Conclusions

MAIT cells in early untreated RA patients are predominantly CD4^+^, express low levels of CD161 and are hyporesponsive upon stimulation compared to SpA patients and controls. Using CD161 as a MAIT cell marker may bias towards exclusion of more poorly functioning MAIT cells. Given the contribution of MAIT cells in the host–microbiome interaction, combined with the notion that the microbiome might contribute to rheumatic diseases, our data showing diminished MAIT cell function in early RA suggest these cells may contribute to dysbiosis in RA.

## Additional files

Additional file 1: **Table S1.** Patient characteristics (PDF 205 kb) (DOCX 24 kb)

Additional file 2: **Figure S1.** Gating strategy. Representative plots showing gating strategy for both PBMCs and SFMCs. Lymphocytes selected based on FSC-A and SSC-A. Doublets removed in both the FSC and SSC, and dead cells excluded based on DAPI for PBMCs and Amcyan (Aqua) for SFMCs. T cells defined within single, live population based on CD3 expression, and MAIT cells gated based on CD3^+^ and MR1-tetramer^+^. MAIT cell subsets defined based on CD4 and CD8 expression as indicated in last panel (PDF 205 kb)

Additional file 3: **Figure S2.** No differences in synovial MAIT cells in treated SpA and RA patients. In contrast to our PBMC samples, all SFMC samples were obtained from patients both with longstanding disease and whom received various treatments. (**A**) SFMC MAIT cells divided based on CD4 and CD8 expression. (**B**) CD161 expression (MFI) of synovial fluid MAIT cells. There were no statically significant differences, neither in MAIT cell subsets nor CD16 expression. (PDF 808 kb)

## Abbreviations

5-OP-RU: 5-(2-Oxopropylideneamino)-6-d-ribitylaminouracil
DAPI: 4′,6-Diamidino-2-phenylindole
DMARD: Disease-modifying anti-rheumatic drug
DN: Double negative
FCS: Fetal calf serum
MAIT: Mucosal-associated invariant T
MFI: Mean fluorescence intensity
MOI: Multiplicity of infection
MR1: MHC class I-related protein 1
NK: Natural killer
PB: Peripheral blood
PBMC: Peripheral blood mononuclear cell
PBS: Phosphate-buffered saline
PMA: 12-O-Tetradecanoylphorbol 13-acetate
RA: Rheumatoid arthritis
SF: Synovial fluid
SFMC: Synovial fluid mononuclear cell
SpA: Spondyloarthritis
