# Supplementary material for: Altered composition and phenotype of mucosal-associated invariant T cells in early untreated rheumatoid arthritis
Source: Arthritis Res Ther. 2019 Jan 5;21:3. doi: 10.1186/s13075-018-1799-1 (PMC6321723; doi:10.1186/s13075-018-1799-1)
Supplement: Supplementary file 1 — Table S1. Patient characteristics (PDF 205 kb) (DOCX 24 kb) [file 13075_2018_1799_MOESM1_ESM.docx]

**Table S1 Patient Characteristics**

|  | RA PBMC*° | AxSpA PBMC** | Controls PBMC^#^ | RA SFMC* | SpA SFMC** |
| --- | --- | --- | --- | --- | --- |
|  | N=10 | N=12 | N=10 | N=8 | N=8 |
| Age median (range), years | 35 (20-38) | 24 (17-39) | 25 (17-39) | 67 (43-82) | 44 (37-54) |
| Males, n (%) | 1 (10) | 7 (58) | 5 (50) | 2 (25) | 3 (38) |
| Median symptoms duration (range),months | 6 (5-36) | 13 (5-24) | 12 (3-24) | 66 (12-516) | 66 (24-192) |
| Current NSAID use, n (%) | 5 (50) | 7 (58) | 6 (60) | 1 (13) | 6 (75) |
| Current DMARD use, n(%) | 0 (0) | 1 (8)^Ŧ^ | 0 (0) | 8 (100) | 3 (50) |
| Current biological use, n(%) | 0 (0) | 0 (0) | 0 (0) | 4 (50) | 2 (25) |
| Rheumatoid and/or ACPA positive, N (%) | 6 (60) | n/a | n/a | 6 (75) | n/a |
| Elevated CRP or ESR, n (%) | 5 (50) | 3 (25) | 2 (20) | 6 (75) | 2 (25) |
| Family history of SpA, n (%) | n/a | 6 (50) | n/a | n/a | 2 (25) |
| Inflammatory back pain, n (%) | n/a | 10 (83) | n/a | n/a | 2 (25) |
| Enthesitis, n (%) | n/a | 2 (17) | n/a | n/a | 0 (0) |
| Good response of back pain to NSAIDs | n/a | 7 (58) | n/a | n/a | 1 (13) |
| Anterior uveitis, n (%) | n/a | 1 (8) | n/a | n/a | 0 (0) |
| Peripheral arthritis, n (%) | n/a | 4 (25) | n/a | n/a | 8 (100) |
| Dactylitis, n (%) | n/a | 1 (8) | n/a | n/a | 2 (25) |
| Psoriasis, n (%) | n/a | 2 (17) | n/a | n/a | 3 (38) |
| IBD, n (%) | n/a | 2 (17) | n/a | n/a | 0 (0) |
| HLA-B27 positive, n (%) | n/a | 8 (67) | n/a | n/a | 3 (38) |
| Sacroiliitis on MRI, n (%) | n/a | 8 (67) | n/a | n/a | 2 (25) |
| Sacroiliitis on X-ray, n (%) | n/a | 2(25) | n/a | n/a | 2 (25) |

RA: rheumatoid arthritis; AxSpA: axial spondyloarthritis; SpA: spondyloarthritis

*all RA patients met the 2010 ACR/EULAR RA criteria; **all patients met the 2009 ASAS criteria for axial or peripheral SpA; ° None of the RA patients used oral steroids; # chronic back pain patients diagnosed with nonspecific back pain (n=7), spinal disc herniation (n=1), scoliosis of the spine (n=1), and hypermobility related back pain (n=1)

Ŧ one patient used low dose methotrexate for psoriasis
