# Supplementary figures and images for: Altered composition and phenotype of mucosal-associated invariant T cells in early untreated rheumatoid arthritis
Source: Arthritis Res Ther. 2019 Jan 5;21:3. doi: 10.1186/s13075-018-1799-1 (PMC6321723; doi:10.1186/s13075-018-1799-1)

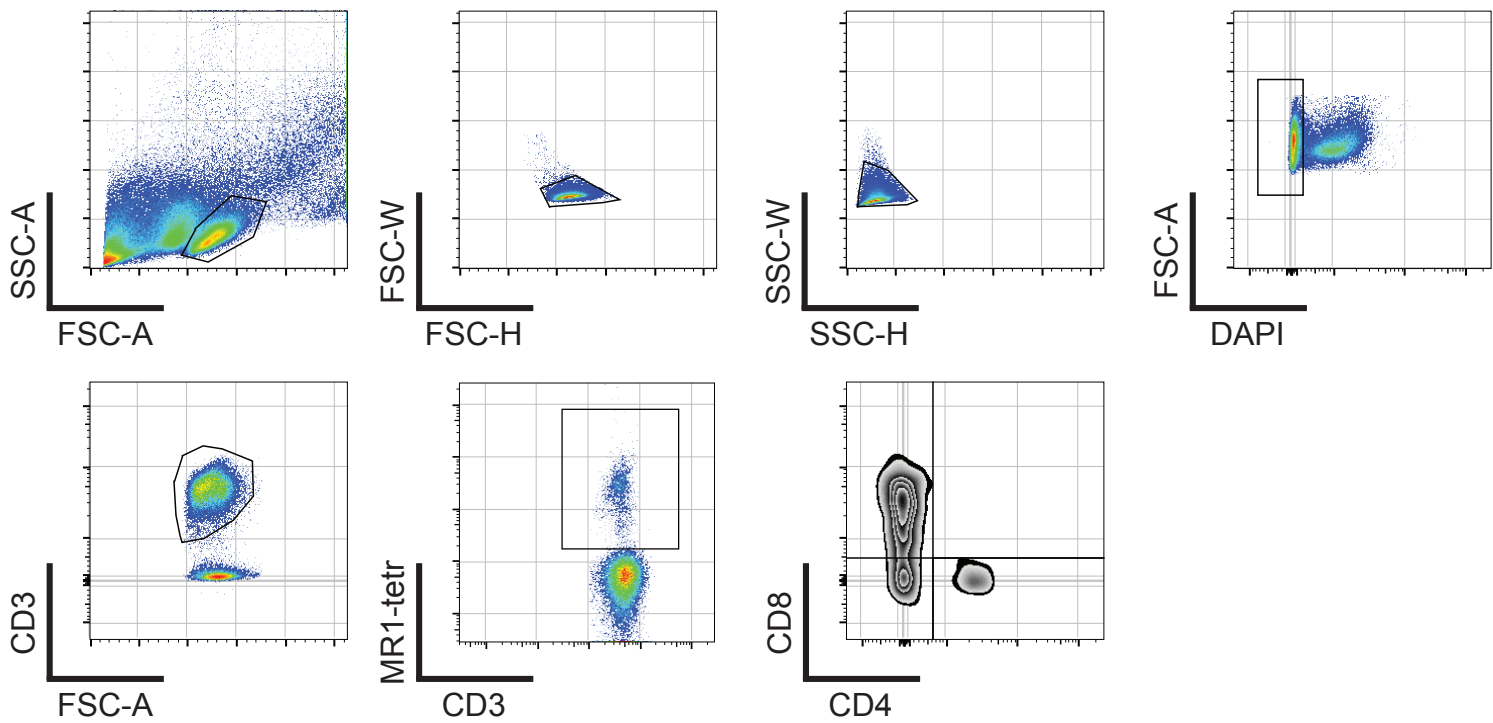

Supplement: Supplementary file 2 — Figure S1. Gating strategy. Representative plots showing gating strategy for both PBMCs and SFMCs. Lymphocytes selected based on FSC-A and SSC-A. Doublets removed in both the FSC and SSC, and dead cells excluded based on DAPI for PBMCs and Amcyan (Aqua) for SFMCs. T cells defined within single, live population based on CD3 expression, and MAIT cells gated based on CD3+ and MR1-tetramer+. MAIT cell subsets defined based on CD4 and CD8 expression as indicated in last panel (PDF 205 kb) [file 13075_2018_1799_MOESM2_ESM.pdf]

A

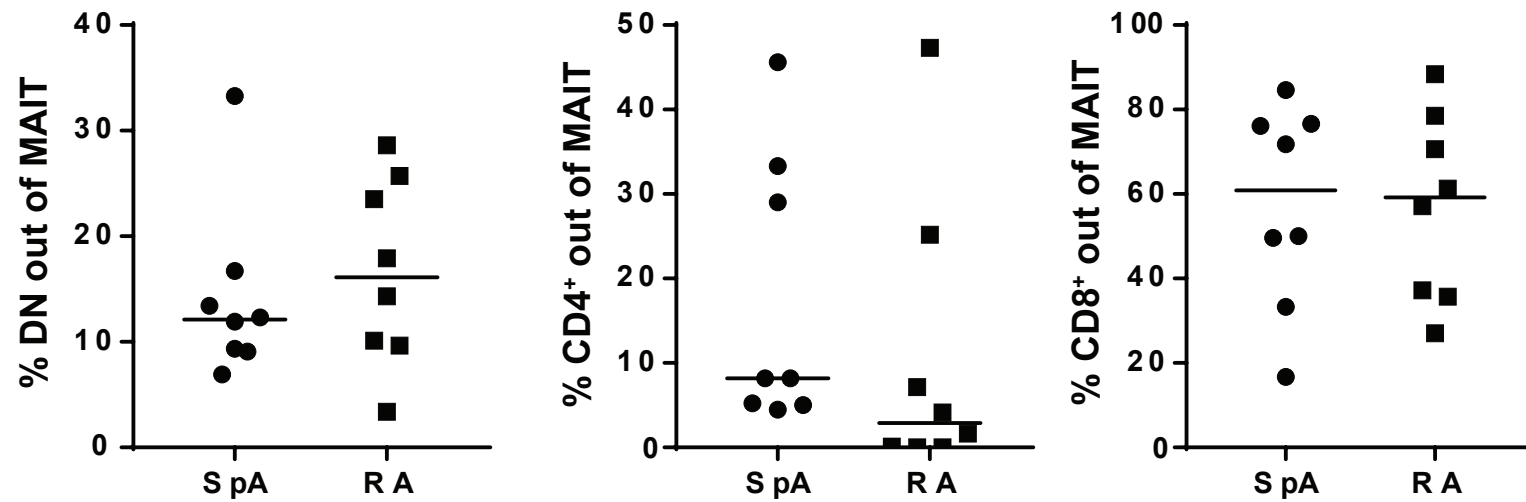

B

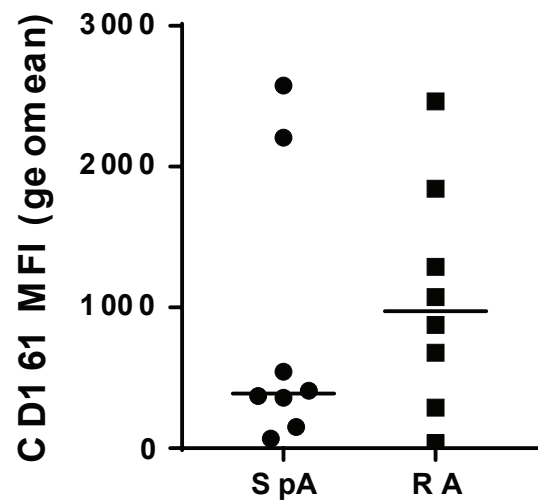

Supplement: Supplementary file 3 — Figure S2. No differences in synovial MAIT cells in treated SpA and RA patients. In contrast to our PBMC samples, all SFMC samples were obtained from patients both with longstanding disease and whom received various treatments. (A) SFMC MAIT cells divided based on CD4 and CD8 expression. (B) CD161 expression (MFI) of synovial fluid MAIT cells. There were no statically significant differences, neither in MAIT cell subsets nor CD16 expression. (PDF 808 kb) [file 13075_2018_1799_MOESM3_ESM.pdf]
